# Biomaterials-mediated ligation of immune cell surface receptors for immunoengineering

**DOI:** 10.1016/j.iotech.2023.100695

**Published:** 2023-12-10

**Authors:** Hao Cui, Lili Zhang, Yang Shi

**Affiliations:** 1 Department of Polymer Therapeutics, Institute for Experimental Molecular Imaging, Uniklinik RWTH Aachen and Helmholtz Institute for Biomedical Engineering, Faculty of Medicine, RWTH Aachen University, Aachen 52074, Germany; 2 Department of Mechanical and Production Engineering, Aarhus University, 8200 Aarhus N, Denmark

**Keywords:** Immune cells, Surface receptors, Crosslinking, Mechanical force, Immunotherapy

## Abstract

A wide variety of cell surface receptors found on immune cells are essential to the body's immunological defense mechanisms. Cell surface receptors enable immune cells to sense extracellular stimuli and identify pathogens, transmitting activating or inhibitory signals that regulate the immune cell state and coordinate immunological responses. These receptors can dynamically aggregate or disperse due to the fluidity of the cell membrane, particularly during interactions between cells or between cells and pathogens. At the contact surface, cell surface receptors form microclusters, facilitating the recruitment and amplification of downstream signals. The strength of the immune signal is influenced by both the quantity and the specific types of participating receptors. Generally, receptor crosslinking, meaning multivalent ligation of receptors on one cell, leads to greater interface connectivity and more robust signaling. However, intercellular interactions are often spatially restricted by other cellular structures. Therefore it is essential to comprehend these receptors' features for developing effective immunoengineering approaches. Biomaterials can stimulate and simulate interactions between immune cells and their targets. Biomaterials can activate immune cells to act against pathogenic organisms or cancer cells, thereby offering a valuable immunoengineering toolset for vaccination and immunotherapy. In this review, we systematically summarize biomaterials-based immunoengineering strategies that consider the biology of diverse immune cell surface receptors and the structural attributes of pathogens. By combining this knowledge, we aim to advance the development of rational and effective approaches for immune modulation and therapeutic interventions.

## 1 Introduction

Malignant tumors typically lead to damage in immune function.^1^ Tumor cells employ diverse mechanisms, such as immune checkpoints and immunosuppressive metabolites, to disrupt the function of anti-tumor immune effector cells.^2^ The restoration and strengthening of immune cell functions are crucial for effectively treating cancer. Over time, a multitude of immunotherapy strategies have been established to regain immunological functions, with some successfully applied in clinical practice. Immune checkpoint inhibitors (e.g., programmed death/ligand 1 (PD-1/PD-L1) and cytotoxic T-lymphocyte-associated antigen 4 (CTLA-4)) and chimeric antigen receptor (CAR)-T cell therapies have made significant progress in extending cancer patients' lives.^3^ Despite these achievements, there are still noteworthy limitations that hinder the effectiveness of these stategies. In particular, CAR-T interventions primarily target hematological cancers and have limited efficacy against solid tumors. Immune checkpoint therapy is mainly used for patients with advanced-stage cancers and benefits only a minority of individuals.^4^ It is still a highly unmet need to understand how to make immunotherapy more effective in non-responsive patients.

An increase in immunoengineering techniques has been brought on by developments in materials science.^5^ For instance, the problem of poor CAR-T cell homing into solid tumors can be efficiently solved by transplanting a 3D scaffold embedded with high-density CAR-T cells into lesions.^6^ Furthermore, nanoparticles coated with checkpoint inhibitors and co-stimulatory molecules achieved spatiotemporal activation of CD8^+^ T cells while minimizing side effects.^7^ The in vivo activation process of immune cells has inspired innovative strategies for immune cell activation. Within the germinal center, B cells engage with follicular dendritic cells (FDCs) to seize antigens using their B cell receptors (BCRs). The subsequent step entails engaging with T helper cells to evaluate and preserve the B cells harboring high-affinity BCRs. This process can be mimicked using liposome-based synthetic germinal centers, inducing germinal center-like B cells and efficient antibody class switching.^8^ To initiate T cell activation, artificial antigen-presenting cells (aAPCs) can accurately replicate the dynamic interplay observed between natural antigen-presenting cells (APCs) and T cells.^9,10^ Nanoscale aAPCs can build up in draining lymph nodes and effectively activate antigen specific T lymphocytes. Moreover, nanoparticles functionalized with CD47-signal regulatory protein α (SIRPα) or Fc receptor (FcR) ligands can balance non-specific phagocytosis and target cell phagocytosis by macrophages.^11,12^ Certain material characteristics have facilitated receptor crosslinking and immune cell activation. For instance, polymers can mimic polysaccharide structures and facilitate multivalent interactions between antigens and receptors.^11^ By mimicking the molecular composition and size of viruses, virus-like particles (VLPs) can effectively elicit immune responses.^13^ Additionally, the stiffness of hydrogels can be adjusted to investigate the transmission of mechanical signals in B cells, thereby enhancing B-cell activation.^14^ By combining surface-functionalized nanoparticles with drug-embedded nanoparticles, immune cells can be simultaneously targeted via both intracellular and extracellular receptors, leading to effective immune priming.^15,16^ Hence, a comprehensive understanding of immune cell activation and the rational design of activation strategy is crucial to achieve effective immune activation.

**Figure 1:**
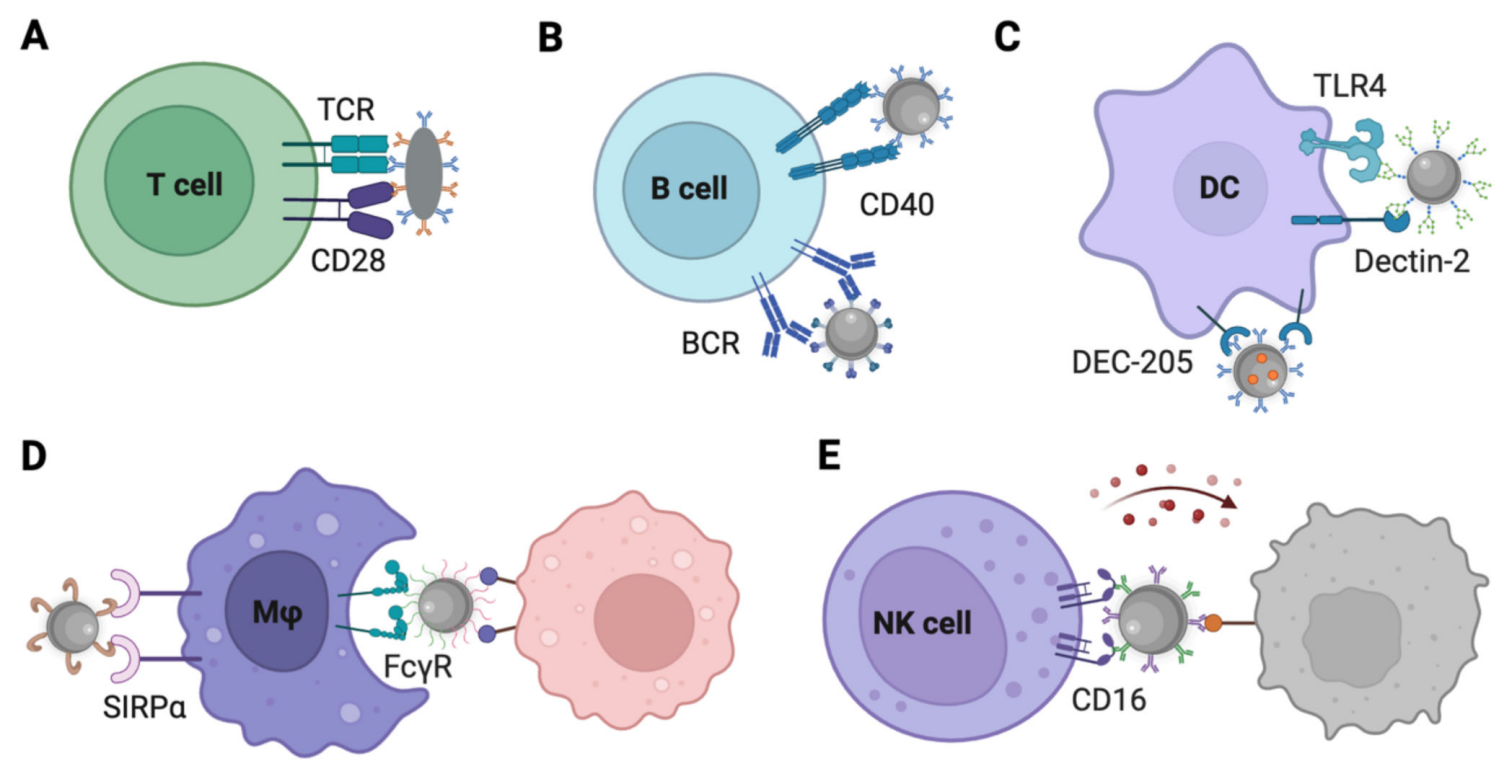
Crosslinking of cell surface receptors induces activation of various immune cells. (A) Artificial antigen-presenting cells (aAPCs) crosslink T cell receptor (TCR) and CD28 to achieve T cells activation. (B) Virus-like particles (VLPs) facilitate the crosslinking of B cell receptor (BCR) and CD40, thereby instigating the activation of B cells. (C) Dendritic cells (DCs) are activated through the crosslinking of pattern recognition receptors (PRRs), including TLR4, Dectin-2, and DEC-205 by multivalent glycans, which form nano-pathogen-associated molecular pattern (PMAP). (D) Macrophages exhibit enhanced phagocytosis when Fcγ receptors (FcγRs) are crosslinked, while the crosslinking of SIRPα inhibits phagocytic activity. (E) CD16 crosslinking activates natural killer (NK) cells for antibody-dependent cellular cytotoxicity (ADCC).

In this review, we outline immunoengineering concepts based on biomaterials, specifically considering the unique characteristics of immune cell surface receptors. As delineated in Fig. 1, these strategies predominantly center around the concept of biomaterial-mediated receptor crosslinking, which sets in motion a series of signaling events and regulatory processes within immune cells. Such interactions are mainly mediated by biomaterials with surface functionalizations. These biomaterials facilitate the crosslinking of cell surface receptors and foster multivalent interactions with antibodies, small molecule antigens, or peptides, thereby maximizing the immune cell activation response.

## 2 T cell surface receptors

T cells, pivotal components of the immune system, depend on a variety of surface receptors to modulate their activation. Among these receptors, the most prominent types are T cell receptors (TCRs), co-stimulatory receptors, and immune checkpoint receptors. TCRs serve the vital function of recognizing antigens and initiating the first step in T cell activation. Co-stimulatory receptors serve the function of diminishing T cell activation thresholds, thus enabling the continuous transmission of activation signals. Conversely, immune checkpoint receptors act as safeguards to prevent excessive T cell activation that may pose a risk to healthy cells. The coordinated interplay of these receptors ensures the maintenance of appropriate levels of T cell activation.

T cell priming by APCs plays an indispensable role in anti-tumor immunity. T cells are activated with multiple stimulation signals, including antigen-TCR engagement, co-stimulation signaling and cytokine ligation with receptors. These signals are provided by APCs during immune reactions, but the efficiency can be quite low. Biomaterials-based aAPCs can mimic the surface receptors of APCs to interact with T cells with an optimized singling intensity. Therefore, aAPCs represent a promising alternative to naturally occurring APCs in the body (Fig. 1A).

### 2.1 TCR

The TCR is the primary receptor that activates T lymphocytes by recognizing the major histocompatibility complex (MHC)-antigen complex (pMHC) presented by APCs.^17^ However, the specific mechanisms by which TCR-pMHC complexes initiate T cell activation remains incompletely understood. A seminal cryo-EM study of a completely constructed TCR-pMHC revealed that there were no significant alterations in the spatial structure of the receptor upon antigen binding.^18^ Intriguingly, TCRs are capable of spontaneously transitioning from an inactive to an active state, even without the presence of antigens.^19^ Notably, the primed TCRs that detach from cholesterol can activate T cells independently of antigen binding. This highlights the crucial role of cholesterol in binding to and stabilizing inactive TCRs, thereby influencing the processes of T cell activation.^19^ Recent evidence suggests that TCR may also function as a mechanosensor, enabling the detection of mechanical force changes at the T cell-APC interface.^17,20,21^ Co-culturing T cells and aAPCs under shaking conditions has been reported to greatly increase T cell activation.^22^ The immunoreceptor tyrosine activation motif (ITAM) is found on CD3, a molecule that the TCR interacts with non-covalently to stabilize its structure and facilitate the transmission of activation signals. T cell activation signal transduction can be stimulated or inhibited by antibodies that target CD3. When the TCR and pMHC interact, a conformational shift in CD3 causes the recruitment of CD4 or CD8,, which in turn stabilizes the connection between MHC II or MHC I and TCR complexes, respectively. LCK, located at the tail of CD4 or CD8, phosphorylates ITAM, subsequently facilitating the recruitment and phosphorylation of ZAP70 by LCK.^17^ ZAP70 further phosphorylates LAT in lipid rafts, promoting the formation of LAT-nucleated signaling complexes that play a critical role in distal TCR signaling.^23^

Resting T cells have TCRs dispersed in a monovalent form across their plasma membranes.^24^ T cell activation is dependent on TCR aggregation via interactions with multivalent pMHC, as monovalent pMHC does not elicit a stimulatory response. Multivalent pMHC induces TCR crosslinking, which promotes the phosphorylation of downstream signaling molecules.^25^ Biomaterials can be surface-functionalized through click chemistry to facilitate TCR crosslinking. Moreover, the combination of primary and secondary antibodies can produce similar effects by transitioning primary antibodies to a multivalently interactive manner. Such multivalent interactions resulting in TCR clustering lead to restricted plasma membrane fluidity and trigger conformational changes in CD3.^17,26^

Various biomaterials have been employed for T cell activate and expansion in vitro and in vivo. The use of aAPCs is the most representative strategy for TCR crosslinking. The ability of aAPCs to crosslink TCRs can be regulated by various factors, including ligand affinity,^27^ crosslinking method,^28^ aAPC size,^29^ scaffold rigidity,^30^ topology,^31^ ligand spacing,^32^ and spatial localization.^33^ The Figdor group developed semi-flexible polymer-based aAPCs to address the limitations of rigid scaffolds that hinder receptor aggregation.^30,34^ By extending the polymer length and density within a specific range, they facilitated multivalent interactions with the TCRs, thereby reducing the threshold to avtivate T cells. In a separate approach, the Schneck group utilized magnetic nanoparticles to control TCR crosslinking using magnetic fields.^28^ The Yu group employed Janus particles to mimic the immune synapse and designed aAPCs with distinct spatial distribution characteristics of ligands.^33^ Their findings revealed that particles with a clustered distribution of anti-CD3 antibodies activated T cells more effectively than those with a uniform distribution of the same antibody amount. Moreover, the anti-CD3 antibody-conjugated matrix serves not only as a scaffold for TCR crosslinking but also as a rheostat for the mechanical force required for membrane deformation.^35,36^ Increasing the substrate stiffness reduces the mechanical force threshold necessary for membrane deformation, thereby enhancing the efficiency of T cell activation. A 15-nm intercellular distance is the best condition for TCR/pMHC interaction.^37^ When considering heightened TCR triggering, the distance of the conjugated TCR ligand relative to the scaffold surface must also be taken into account.^38^ To investigate the spatial demands for T cell activation, the Sevcsik group developed a DNA origami-based biointerface.^39^ Their findings demonstrated that antigen-mediated T cell activation was not strongly influenced by the spatial arrangement of pMHC.

T cell signaling and activation are significantly influenced by the mechanical procedures by which TCRs recognize antigens and interact with pMHCs at the molecular level. Multiple experimental studies have aimed to comprehend T cell sensitivity and selectivity toward antigens, as well as to characterize the structural and biophysical features of TCR-pMHC interactions.^40,41^ During antigen scanning, the tension on the TCR-pMHC bond influences T cell antigen discrimination.^42^ The Dushek group suggested that tensile forces impeded this discrimination while force-shielding mediated by costimulatory and adhesion molecule in the immunological synapse promoted it.^43,44^ TCRs exert pulling forces of up to 5 pico-newtons on antigens as T cells move, which is sufficient to break the bonds between TCRs and antigens.^45^ The Schütz group developed antibody-coated beads targeting CD3 and CD28 to directly measure the 3D traction forces generated by single microvilli during T cell activation.^42^ Their findings revealed that higher Ca2+ levels, which indicated stronger T cell activation, were associated with increased force from shifts in substrate stiffness.^42^ Molecular dynamics simulations were employed to explore the formation of catch-or slip-bonds during TCR-pMHC disengagement, highlighting that even little variations in the TCR-pMHC interface may exert considerable disparities in signaling outcomes.^46,47^ The Garcia group devised a TCR-pMHC catch bond engineering strategy to generate high-potency, low-affinity TCRs with reduced risk of off-target toxicity in immunotherapy.^48^ Additionally, electrostatic interactions between the receptor and the membrane contributed to the regulation of TCR signaling. The Gaus group has mapped membrane charges and analyzed TCR clustering in living T cells, providing insights into the role of membrane charge in various cellular activities.^49^

### 2.2 Co-stimulatory receptor

T cell activation is dependent on the collaboration of TCRs and co-stimulatory receptors on the surface of T cells. Co-stimulatory receptors and ligands interact at the immunological synapse once the TCR recognizes pMHC, where they contribute to downstream effector molecule phosphorylation, cytoskeleton remodeling, and robust transcription of effector gene.^50,51^ CD28 is a well-studied co-stimulatory receptor that interacts with B7 ligands (CD80 and CD86) on APC membranes.^52^ Recent studies have unveiled a distinctive form of T cell self-activation induced by B7 proteins produced by T cells or acquired from APCs. In this process, T cells contract their membrane inward, facilitating the binding of B7 and CD28. This mechanism boosts CD8^+^ T cell priming and promotes anti-tumor immune responses.^53^ Fab fragments of anti-CD28 antibodies are unable to crosslink CD28 and, therefore, cannot activate T cells. Conversely, intact anti-CD28 IgG can form multivalent complexes by interacting with anti-IgG antibodies or FcRs, promoting CD28 crosslinking and T cell activation.^54^ This approach can also be extended to other co-stimulatory receptors such as OX-40,^55,56^ ICOS,^57,58^ 4-1BB,^59^ GITR,^60^ and CD96.^61,62^ It is worth noting that bispecific antibodies can serve as scaffolds for receptor crosslinking. For instance, Chan et al. demonstrated that bispecific agonists targeting both PD-1 and GITR induced aggregation and signaling of GITR in a PD-1-dependent manner.^60^ Furthermore, Soldevilla et al. reported that a tumor-targeted MRP1-ICOS bispecific aptamer could attach to tumor cell membranes for an extended period, facilitating more efficient crosslinking of ICOS.^58^

**Figure 2:**
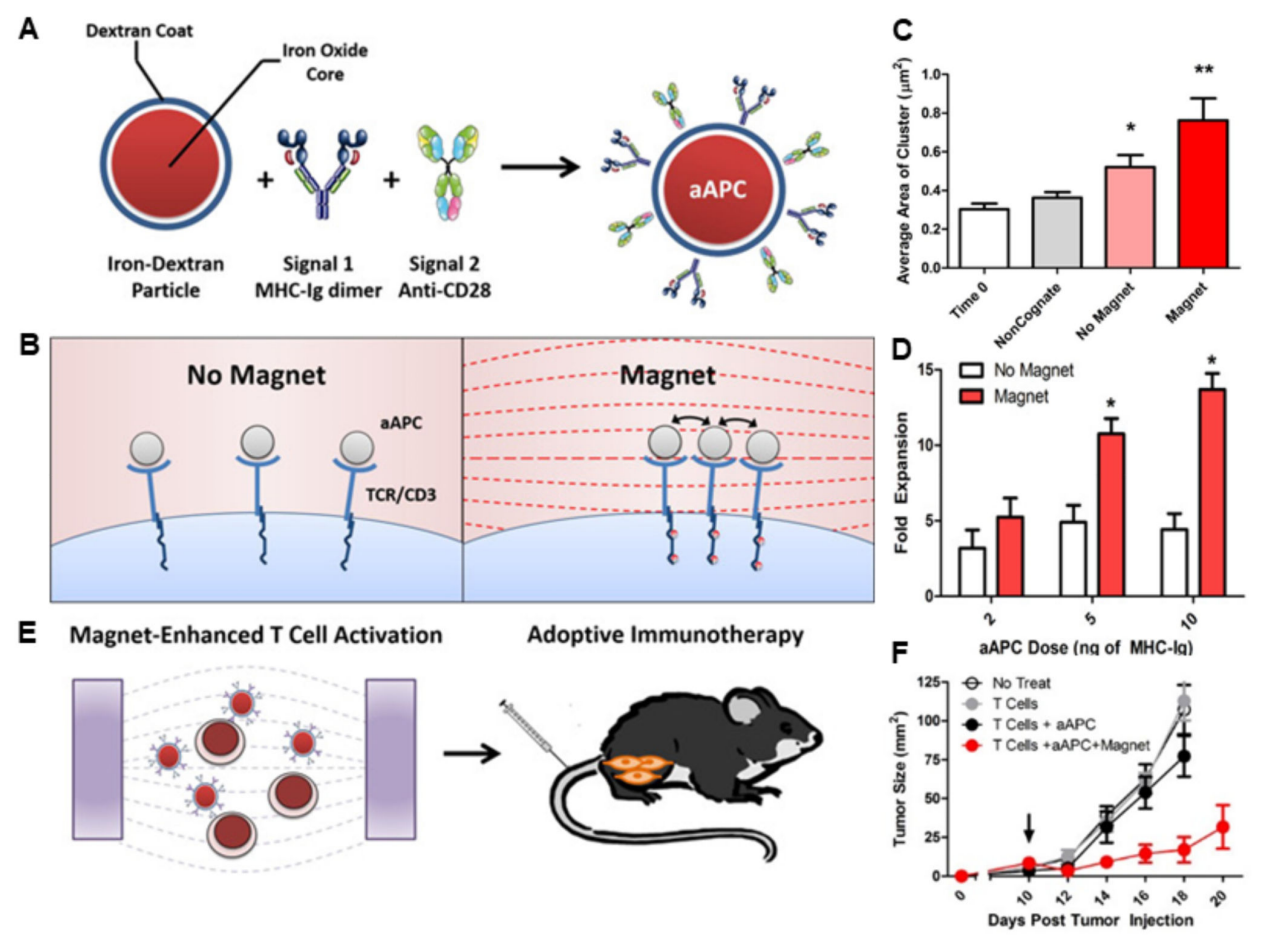
Magnet-mediated TCR crosslinking enhances CD8^+^ T cell-mediated anti-tumor efficacy. (A) Structure of artificial antigen presenting cells (aAPCs). (B) TCR crosslinking mediated by aAPCs using magnets. (C) Magnet-promoted TCR cluster formation. (D) Expansion of Pmel T cells following aAPCs treatment, with or without magnets. (E) CD8^+^ T cells were activated by aAPCs in the presence of a magnet before proceeding with adoptive immunotherapy. (F) Anti-tumor responses in CD8^+^ T cells activated with aAPCs and magnets. A-F are adapted from Perica et al. ^28^

Various biomaterials have been employed to develop aAPCs for antigen-specific T cell expansion in vitro or directly activate T cells in vivo.^63^ aAPCs are generally coupled with ligands for TCRs and co-stimulatory receptors. aAPCs-mediated receptor crosslinking can be modulated through diverse methods, such as particle size, shape, and material type. Contrary to previous findings suggesting that nanoscale aAPCs were ineffective in receptor crosslinking due to their large curvature, the Schneck group reported that nanoscale aAPCs actually facilitated receptor crosslinking and T cell expansion.^9^ They attributed this success to the utilization of the MHC-Ig dimer, which had a flexible hinge region capable of efficiently crosslinking TCRs. Nanoscale aAPCs, with diameters ranging from 50 to 100 nm, exhibit efficient distribution within lymph nodes and tumors, while microscale aAPCs predominantly localize at the injection site. Additionally, nanoscale aAPCs offer unique advantages. For example, ligands of TCRs and co-stimulatory receptors can be conjugated to paramagnetic nanoparticles (Fig. 2A),^28^ enabling the control of TCR and CD28 crosslinking through a magnetic field. (Fig. 2B). Furthermore, magnetic fields significantly enhanced the TCR aggregation mediated by nanoscale aAPCs, thereby promoting T cell expansion (Fig. 2C, 2D). Conversely, microscale aAPCs are relatively large and cannot effectively crosslink receptors in magnetic fields. To expand tumor antigen-specific T cells, Trp2-loaded aAPCs were utilized and subsequently transferred into tumor-bearing mice (Fig. 2E). This approach exhibited significant inhibition of melanoma growth (Fig. 2F).

Both the material and the shape of nanoparticles significantly influence receptor crosslinking. When ligands of TCRs and co-stimulatory receptors are dispersed on fluid cell membranes, they have the flexibility to move, aggregate, and form immunological synapses.^17^ However, conjugated-ligands on rigid nanoparticles showed impeded receptor aggregation.^64^ In contrast, flexible materials promote aggregation during receptor-ligand interactions. For example, semi-flexible polymers coupled with anti-CD3/anti-CD28 antibodies facilitated the crosslinking of CD3 and CD28, surpassing the T cell activation achieved by ligand-functionlized rigid PLGA nanoparticles.^64^ To overcome the limited fluidity of rigid nanoparticles, PLGA nanoparticles coated with cell membranes or lipid bilayers emulated the fluidic nature of cell membranes, thereby facilitating receptor crosslinking.^65^ The amount of receptor crosslinking depends critically on the surface area of interaction between nanoparticles and cells. Therefore, elliptical, rod-shaped nanoparticles, or nanotubes with high aspect ratios are more efficient in promoting receptor crosslinking.^66–68^ Furthermore, incorporating adhesion molecules like LFA-1 and ICAM-1 establishes a stable connection between aAPCs and T cells, which improves CD3 and CD28 crosslinking on T cells.^69,70^.

## 3 B cell surface receptors

B cells are essential in humoral immunity and can potentiate cellular immunity. The potential importance of B cells in cancer has been a long debate, B cells are also often recognized as regulatory B cells in tumors but increasing recent evidence demonstrated that B cells can induce potent therapeutic effects for cancer.^71^ B cells, especially located in intratumoral tertiary lymphoid structures (TLS), can effectively provoke cancer immunity. Furthermore, B cells in draining LNs can interact with helper T cells to provoke cancer immunity. In the context of engineering B cell immunity for cancer therapy, biomaterials can provide multiple B cell activation signals, including BCR crosslinking and CD40 engagement (Fig. 1B). In addition, B cell immunity can be combined with other therapeutics modalities such as checkpoint blockade therapy and chemotherapy.

### 3.1 BCR

BCRs present on the surface of B cells are mainly responsible for identifying invading pathogens. The BCR consists of a membrane-bound immunoglobulin (mIg) and the Igα/Igβ heterodimer.^72^ The 3D molecular structure of the BCR was discovered by cryo-electron microscopy, demonstrating that the symmetrical mIg binds solely to Igα and Igβ on one side to produce an asymmetrical complex.^73^ On resting B cells, the BCR forms ordered oligomers comprising multiple subunits, which remain inactive.^74^ The BCR plays a vital role in recognizing and internalizing antigens, marking an early event in B cell activation. Defects in BCR signaling can result in immunodeficiency. Increasing the number of BCRs during the maturation of B cells can restore the function of immunodeficient B cells.^75^ The Gold group discovered that Toll-like receptor ligands can enhance BCR signaling by blueucing the receptor's spatial limitation caused by actin dependence.^76^ This increase in BCR mobility and collisions at the membrane triggers signaling within the cell, even in the absence of antigen.

Antigens are physically internalized from cell surfaces by B cells via mechanical forces. The characteristics of the antigen, including valence and affinity, significantly impact B cell activation, plasma cell differentiation, and affinity maturation. Multivalent antigens are more easily internalized by the BCR than monovalent antigens. The internalized antigens are degraded into peptide fragments and assembled with MHC II, which could be recognized by CD4^+^ T cells.^77^ Antibody production is dependent on BCR-mediated uptake of high-affinity antigens.^78^ BCR internalizes the antigen through the contraction and pulling of actin cytoskeleton. During this process, bonds formed between the BCR and monovalent or low-affinity antigens are easily disrupted. BCR crosslinking facilitated by membrane-bound high-affinity antigens is sufficient for B cell activation.^78^ Additionally, adhesion molecules between cell contact surfaces can enhance BCR ligation with low-affinity antigens.^79^ B cells can also sense the stiffness of antigen-presenting substrates through the BCR.^80^ This is showcased by that germinal center B cells receive antigens presented by FDCs with relatively stiff cell membrane, thereby germinal center B cells need to exert relatively high mechanical forces when extracting antigens from FDCs. Memory B cells also exhibited higher traction forces to capture membrane-bound antigens compared to naïve B cells.^81^

**Figure 3:**
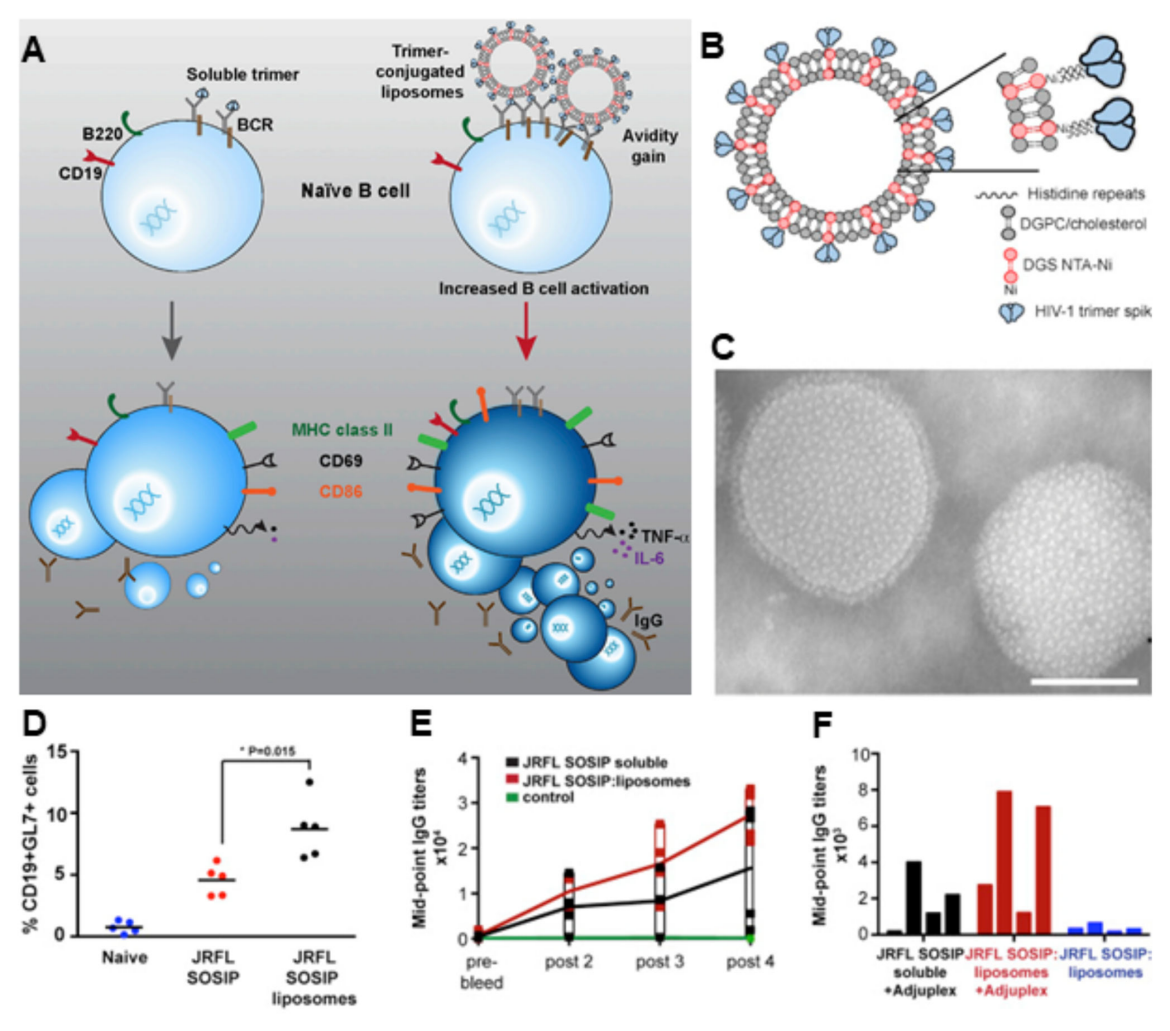
Synthetic liposomes modified with HIV-1 trimer spikes for B cell activation. (A) Schematic of B cell activation through HIV-1 trimer spike-conjugated liposomes. (B) Schematic of the liposome structure utilized for displaying HIV-1 trimer spikes. (C) Representative image of JRFL SOSIP trimer-conjugated liposome. Scale bar, 100 nm. (D) Proportion of germinal center CD19^+^GL7^+^ B cells following treatment with JRFL SOSIP trimer in a soluble form or conjugated form. (E) Production of IgG after treatment with JRFL SOSIP trimer in a soluble form or conjugated form. (F) IgG production following JRFL SOSIP trimer-conjugated liposome treatment, with or without TLR7/8 agonist R848. A-F are adapted from Ingale et al.^13^

B cell activation can be strongly induced by multivalent antigen-mediated BCR crosslinking.^82–84^ Despite monovalent antigens are capable of activating B cells, they cannot be efficiently internalized by BCRs.^77^ The crosslinking of BCRs allows Lyn to phosphorylate Igα/Igβ ITAM, leading to Syk recruitment.^85^ This cooperative action between Lyn and Syk enables a rapid response to BCR clustering and initiates the BCR signaling cascade. Although the exact conformational changes during BCR crosslinking are still unknown, the use of multivalent antigens to activate B cells has been well-established. Virus particles effectively induce B cells to generate specific antibodies because they have repeating epitopes on the outer layer that facilitate BCR crosslinking.^86^ VLPs mimic this physical structure by displaying polymeric epitopes on their surfaces, resulting in stronger activation compared to soluble antigens.^13^ This strategy not only enhanced antigen affinity for better uptake by APCs but also allowed for modulation of VLP size to improve lymph node drainage.^87,88^ VLPs with sizes ranging from 50 to 100 nm were easily absorbed onto the surface of FDCs, facilitating antigen presentation to cognate B cells.^89^ Envelope glycoproteins are predominantly displayed on the surface of virus particles. In the study conducted by Ingale et al., DGS-NTA(Ni) liposomes were developed to present JRFL SOSIP trimers, an HIV-1 envelope glycoprotein mimic, with a C-terminal His-tag (Fig. 3A, 3B).^13^ Negative stain cryo-electron microscopy revealed a uniform arrangement of JRFL SOSIP trimers on the liposome surface (Fig. 3C). JRFL SOSIP trimer-conjugated liposomes exhibited a higher induction of B cell activation markers compared to soluble form in vitro. Additionally, JRFL SOSIP trimer-conjugated liposomes improved the in vivo formation of germinal center (Fig. 3D) and boosted the generation of high-affinity antibodies (Fig. 3E). Further enhancement of IgG production was achieved by mixing the TLR7/8 agonist R848 with JRFL SOSIP trimer-conjugated liposomes (Fig. 3F). DNA origami scaffold-based VLPs promoted BCR signaling and antibody generation against SARS-CoV-2 via a titer-dependent manner by displaying multivalent antigens.^90^ These studies highlight the significance of nanoparticle-mediated multivalent antigen display for BCR crosslinking and the induction of effective humoral immunity.

Polysaccharide vaccines have made remarkable contributions to the management of Streptococcus pneumonia, Neisseria meningitidis and Haemophilus influenzae type b.^91^ These vaccines exploit bacterial surface polysaccharides, which consist of repeating carbohydrate units, to induce B cell expansion and antibody production via BCR crosslinking.^92^ However, traditional polysaccharide vaccines cannot be recognized by TCRs. Consequently, polysaccharides-activated B cells didn’t undergo class switching and differentiate into memory B cells. Therefore, traditional polysaccharide vaccines can only enable B cells to produce low-affinity antibodies.^93^ To overcome these limitations, polysaccharides have been conjugated to antigens containing T-cell epitopes. In vivo studies have revealed that germinal center B cells acquired glycoconjugate vaccines from FDCs through BCR interactions. This led to CD4^+^ T cell response.^92^ Avci et al. conducted a detailed study that shed light on this process.^94^ To crosslink BCRs, they conjugated T cell-independent group B streptococcal type III (GBS III) polysaccharide to ovalbumin (III-OVA) or ovalbumin peptide (III-OVAp). III-OVA was uptook by B cells and processed into glycan-peptides, which were then presented to CD4^+^ T cells. The processed glycan-peptide-MHC II complex directly mediated B cell-T cell interaction, whereas the processed glycan alone did not participate in this process. The production of IL2 and IL4 by CD4^+^ T cells promoted B cell to proliferate and generate GBSIII-specific IgG antibodies. Compared to III-OVA, III-OVAp induced stronger OVA-specific IgG titers and provided protection against Streptococcus in mice. Increasing the amount of GBSIII per unit III-OVAp further enhanced the immunogenicity of the vaccine. Taking inspiration from the design principles of polysaccharide vaccines, Bennett et al. modified polymers with B cell antigens and T cell antigens, with T cell antigens conjugated using enzyme-sensitive linkers.^95^ Modified polymers containing both epitopes evoked stronger antibody responses than those containing only B cell antigen epitopes. T cell antigens conjugated via enzyme-sensitive linkers were more efficiently processed and assembled with MHC II, resulting in enhanced B cell activation compared to those conjugated via enzyme-insensitive linkers. These studies provide valuable guidance for developing glycan-based vaccines.

BCR is a mechanosensitive receptor that has been extensively studied.^14,81,96–98^ When B cells encounter antigens, their cytoskeletal proteins undergo remodeling, causing membrane-bound BCR-antigen complexes to spread and aggregate. The generated mechanical force disrupts the weak bonds between the BCR and antigen while retains multivalent antigen-BCR complexes to form microclusters.^78^ These microclusters are essential for transducing mechanical force and initiating the phosphorylation of Igα/Igβ ITAM.^99^ However, the precise mechanism by which mechanical force-induced conformational changes in the BCR complex facilitate ITAM phosphorylation remains unknown.^100^ Spillane et al. demonstrated that B cells exhibited varying responses when mechanically extracting antigens from the APC's surface, and these responses were influenced by the stiffness of the APC.^80^ This finding suggests the exciting possibility of actively controlling B cell responses and antibody production by manipulating the physical cues within the immune synapse. To explore how mechanical forces affect B cell development, hydrogels based on polyacrylamide and polydimethylsiloxane have been used as models.^14^ The Liu group reported that B cells formed larger BCR microclusters on stiffer antigen-tethered hydrogels.^97^ The change in physical stiffness recruited pSyk and pTyr to the microclusters, resulting in enhanced CD69 expression.^14,98^ It's crucial to highlight that B cells exhibit different sensitivities to mechanical forces at different stages of development.^81,96^ The generation and transmission of mechanical force rely on extensive BCR crosslinking, similar to how adhesion molecules convert relative movement between cells or cell-matrix interfaces into mechanical forces. Adhesion molecules strengthened the binding between the BCR and antigens, enabling the discrimination of substrates with varying stiffness.^96^ Antigens with low affinity for B cells exhibited higher efficiency when presented on a soft surface.^80^ In certain diseases, like rheumatoid arthritis, matrix stiffness also impacts the pathological changes of B cells.^81^

These exciting findings promote the recognition of mechanical forces in imunotherapy.^101^ For example, mechanical forces could enhance cancer killing by cytotoxic T cells.^102^ Also, mechanosensing may affect B cell response to viral infection and the immune response.^99^ The presence of substantial amounts of B cells in the tumor microenvironment underscores their active role in shaping tumor immunity. Leveraging B cells' mechanosensitivity may have significant implications for cancer immunoengineering.

### 3.2 CD40

B cell proliferation and activation depend on three stimuli: BCR ligation, CD40 ligatioin, and cytokines.^103^ BCR is primarily activated by antigens, while CD40 signaling and cytokines (IL4, IL21) mainly originate from CD4^+^ T cells.^104^ CD40 crosslinking on B cell’s surface enables antibody class switching as well as generating high-affinity antibody, while B cells activated via T cell-independent pathway can only produce low-affinity antibodies. Multivalent CD40 crosslinking significantly improves the activation of B cells. Free anti-CD40 antibodies can only be crosslinked by FcR when cells expressing FcR come into contact with B cells.^105^ This reflects the spatial limitation of free anti-CD40 antibodies in activating B cells. The multivalent display of anti-CD40 antibodies by nanoparticles or VLPs has been developed to activate B cells.^106,107^ The B cell activation efficiency of luminescent porous silicon nanoparticles-conjugated anti-CD40 antibodies was 30-40 times higher than that of free CD40L.^107^ Furthermore, the half maximal effective concentration of P22 virus-like particles-conjugated CD40L was 53.6 times lower than that of free CD40L for B cell activation.^106^ Engineered cells expressing CD40L have been used for CD40 crosslinking and B cell activation in 2D or 3D culture models.^8,108^

## 4 Dendritic cell surface receptors

DCs are among the most important APCs, characterized by a series of PRRs on their plasma membrane.^109^ The PRRs mediate antigen internalization and DC activation. Internalized antigens undergo processing into peptide sequences which are loaded in MHC I or MHC II and are then presented to CD4+ and CD8+ T cells. PRR crosslinking effectively induces DC activation and antigen presentation functions. PRR-targeting antibody- or polysaccharide-modified nanoparticles can be efficiently drained to lymph nodes and taken up by DCs to initiate anti-tumor immune response (Fig. 1C).^110,111^

### 4.1 C-type lectin receptor (CLR)

DCs express CLRs that are essential for antigen recognition and internalization.^112^ These receptors primarily recognize carbohydrates and glycosylated antigens on pathogen surfaces.^113^ Given that bacteria and viruses are covered with glycans, the recognition of glycans and glycosylated antigens by CLRs is a crucial step in recognition of pathogens by DCs.^114,115^ Notable CLRs include DC-SIGN, mannose receptor, and DEC-205. While glycans can mediate antigen recognition and internalization, natural glycans generally exhibit low affinity for CLRs.^116^ Increasing the valence of glycans enhances their affinity for CLRs and facilitates CLR crosslinking-mediated antigen internalization.^117^ The Kooyk group coupled DC-SIGN targeting glycans with OT-I or OT-II epitopes to poly(amido amine) (PAMAM) dendrimers, achieving effective DC-SIGN-dependent delivery of the OT-I or OT-II epitopes.^117^ The internalized antigens were found to localize to lysosomes. Interestingly, high multivalent PAMAM dendrimers activated CD4^+^ T cells, whereas low multivalent PAMAM dendrimers appeared more favorable for activating CD8^+^ T cells. It should be emphasized that DC-SIGN signaling alone was insufficient to induce DC maturation; a second signal, Toll-like receptor 4 (TLR4), was also required. Through the synergistic effect of DC-SIGN and TLR4, DCs were activated by multivalent glycans, leading to IL-10 secretion. This effect was enhanced with an increase in glycans valency. TLR4 additionally guided antigens into the cytoplasm, where they were processed via the proteasome pathway and loaded on MHC I.^118,119^ The Figdor group reported that surface decoration of nanoparticles with anti-DC-SIGN antibody hD1 effectively reduced the non-specific phagocytosis of nanoparticles in vivo, facilitating antigen delivery to DCs.^120^ They compared nanoparticles modified with ICAM3, another natural ligand of DC-SIGN, to those modified by hD1.^121^ hD1 exhibited higher affinity for DC-SIGN than that of ICAM3. Consequently, hD1-conjugated nanoparticles were more efficiently internalized by DCs than their ICAM3 counterparts. However, ICAM3-modified nanoparticles preferentially activated CD8^+^ T cells. Interestingly, high-affinity nanoparticle-mediated antigen internalization appears to have a detrimental effect on antigen cross-presentation, yet the exact mechanism behind this phenomenon remains unclear.

Ligand-functionalized nanoparticles that are recognized by DEC-205 or the mannose receptor have shown significant potential in enhancing antigen presentation and cross-presentation by DCs.^122–124^ Bandyopadhyay et al. decorated anti-DEC-205 antibodies on OVA-loaded PLGA nanoparticles.^123^ Nanoparticles coated with different density of anti-DEC-205 antibodies resulted in different degrees of DEC-205 crosslinking, exhibiting a proportional relationship with the Th2 response while not significantly promoting the Th1 response. Furthermore, DCs' production of IL-10 was associated with the density of anti-DEC-205 antibodies. Scavenger receptor CD36 was also upregulated by the functionalized nanoparticles. IL-10 production was inhibited by CD36 blockage, indicating a potential link to CD36-mediated phagocytosis of apoptotic cell debris.^125^ Polyanhydride nanoparticles decorated with dimannose facilitated the internalization of nanoparticles by crosslinking mannose receptors, leading to DC maturation.^124^ Crosslinking of the mannose receptor predominantly increased IL-6 and TNF-secretion, without detectable secretion of IL-10, which differed from the response observed with DEC-205 crosslinking. Zhang et al. reported that alginate nanoparticles were decorated with mannose and conjugated with OVA using a pH-sensitive linker.^122^ These functionalized nanoparticles demonstrated a significant enhancement in antigen cross-presentation and effectively inhibited EG7 tumor growth. These findings indicate that promoting CLR crosslinking appears to be a promising strategy for enhancing antigen uptake and presentation by DCs.

### 4.2 TLR4

**Figure 4:**
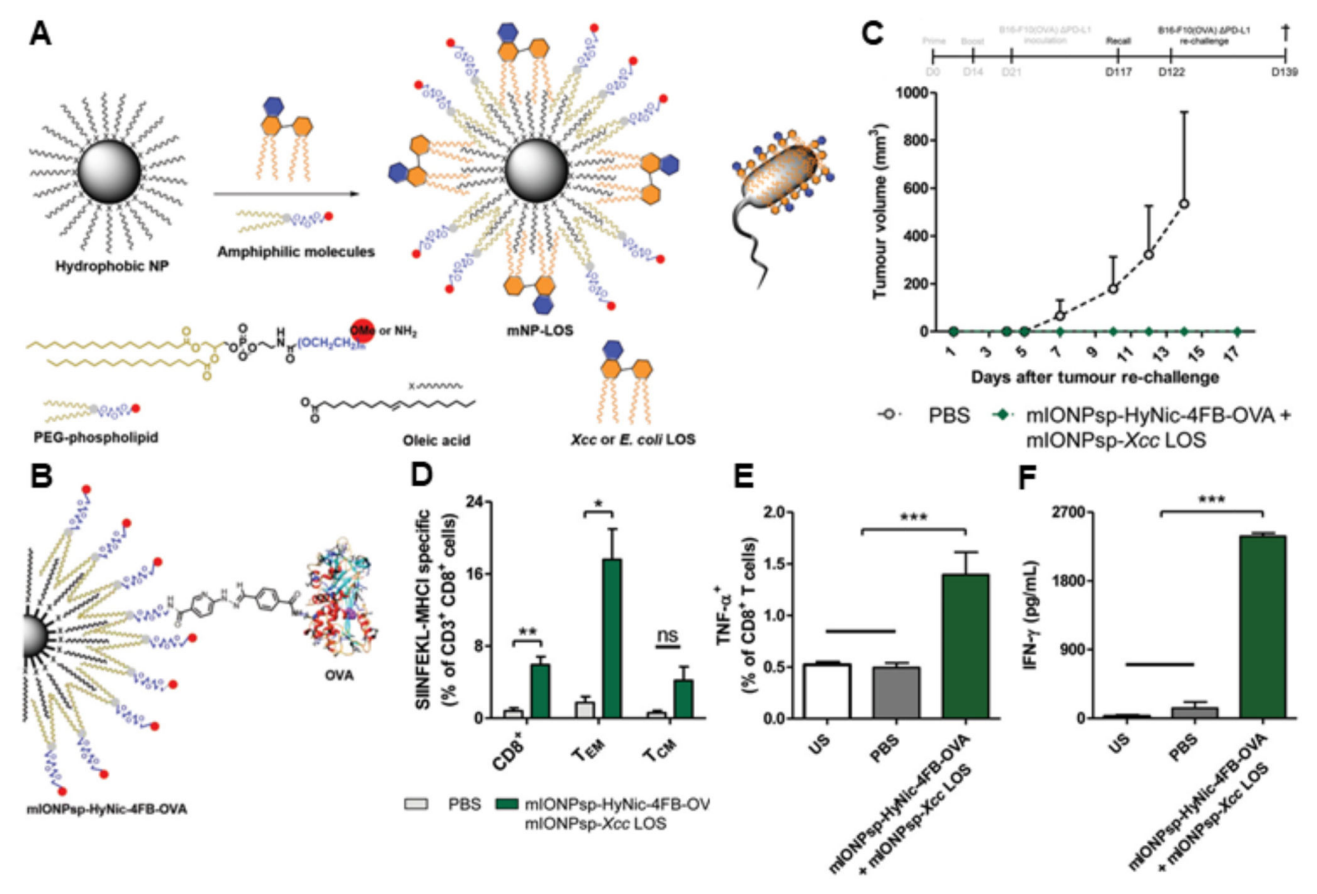
Pathogen-like nanostructures for activation of DCs. (A) Schematic of mIONPsp-*Xcc* LOS synthesis. (B) Structure of mIONPsp-HyNic-4FB-OVA. (C) Anti-tumor effect in B16-F10(OVA) ΔPD-L1 tumor re-challenge with mIONPsp-Xcc LOS and mIONPsp-HyNic-4FB-OVA treatment. (D) Proportion of central memory T cells (Tcm) and effector memory T cells (Tem) after the recall immunization in (C). (E, F) Assessment of TNF-α^+^ CD8^+^ T cells proportion (E) and IFN-γ secretion (F) after in vitro SIINFEKL treatment of immunized mouse splenocyte mixtures. A-F are adapted from Traini et al.^129^

TLR4 signaling and antigens work together to facilitate the maturation of DCs by promoting downstream signaling activation, such as NF-κB.^117,126^ While lipopolysaccharide (LPS) is the natural ligand for TLR4, LPS monomers alone have been shown to be less effective than multivalent TLR4 for DC maturation. Amphiphilic LPS self-assembles into aggregates above a critical concentration in aqueous phase. Notably, at the same concentration of LPS, only LPS aggregates rather than LPS monomers promoted TLR4 activation.^127^ Bacteria possess multivalent LPS that enhances their affinity to TLR4 and mediates TLR4 clustering.^128^ To mimic this bacteria-like structure, researchers employ nanoparticles coated with a hydrophobic layer of LPS to study the kinetics of TLR4 activation.^127^ In the study by Traini et al., Xanthomonas campestris pv. campestris (Xcc) lipooligosaccharide (LOS), a specific type of LPS without the O-antigen chain, was utilized as an immune adjuvant. LOS was adsorbed onto superparamagnetic iron oxide nanoparticles (IONPsp) through hydrophobic interactions, creating a pathogen-like nanostructure called mIONPsp-Xcc LOS (Fig. 4A).^129^ Additionally, the formation of mIONPsp-HyNic-4FB-OVA occurred through the linkage of the model antigen OVA to mIONPsp using a hydrazone bond (Fig. 4B). This functionalized nanoparticle offered the advantage of targeted delivery to the lymph nodes. In comparison to Xcc LOS alone, mIONPsp-Xcc LOS not only enhanced IL-6 secretion by DCs but also reduced cytotoxicity. mIONPsp-Xcc LOS worked with mIONPsp-HyNic-4FB-OVA to induce superior anti-tumor efficacy compared to the combination of Xcc LOS and OVA. Notably, It markedly raised the proportion of memory CD8^+^ T cells that are specific for SIINFEKL and induced robust anti-tumor memory (Fig. 4C, 4D). A mixture of splenocytes from immunized mice exhibited a stronger response in vitro to the OVA epitope SIINFEKL, promoting TNF-α^+^ CD8^+^ T cell proportions and IFN-γ secretion (Fig. 4E, 4F). In another study by Son et al., Saccharomyces cerevisiae mannan-based nanocapsules (Mann-NC) were developed to crosslink Dectin-2 and TLR4 in DCs, which in turn promoting DC maturation and pro-inflammatory cytokines secretion.^130^ Additionally, Mann-NC induced CD4^+^ T cells to differentiat into T_H_17 cells. When Mann-NC was intratumorally injected, it remodeled the tumor immune microenvironment and elevated the T_H_17/Treg ratio. Furthermore, CD8^+^ T cells and NK cells were attracted by T_H_17-secreted cytokines to inhibit the growth of the CT26 tumor.

## 5 Macrophage surface receptors

Macrophages possess robust phagocytic capabilities for eliminating foreign and pathogenic substances within the body. Dysregulated phagocytosis of macrophages hinders the removal of pathogens, potentially exacerbating immune imbalances. Furthermore, alternatively activated macrophages (M2) inhibit cancer immunity and immunotherapy. Maintaining anti-cancer phonotypes of macrophages is paramount for effective tumor eradication.

### 5.1 SIRPα

SIRPα belongs to a class of inhibitory receptors that regulate phagocytosis in innate immune cells.^131^ SIRPα primarily interactes with CD47 and this interaction is crucial in maintaining internal homeostasis by preventing macrophages from engulfing healthy cells. CD47 binds to SIRPα with low affinity and inhibits phagocytosis by hindering myosin assembly.^132^ Cancer cells express CD47 to disrupt macrophage phagocytosis. To restore macrophage phagocytosis of cancer cells, it is essential to block the SIRPα-CD47 interaction. Unfortunately, systemically delivering anti-CD47 antibodies generally induces macrophages to mistakenly phagocytose normal red blood cells and platelets, leading to severe anemia. Therefore, the development of safe strategies that effectively disrupt the CD47-SIRPα signaling holds great potential for cancer treatment.^131^ Ha et al. discovered that the accumulation of SIRPα in lipid microdomains is a prerequisite for the activation of CD47-SIRPα signaling.^133^ These lipid rafts are predominantly found in non-apoptotic cells, facilitating the multivalent binding of CD47 and SIRPα to prevent phagocytosis.^134^ During apoptosis, CD47 diffusion weakens the low-affinity connection between CD47 and SIRPα to promote phagocytosis by macrophages. These findings highlight the significance of the multivalent binding of CD47 and SIRPα in CD47-SIRPα signaling. Rodriguez et al. conjugated CD47-self peptides to VLPs, which effectively inhibited myosin-II-mediated contractility and reduced macrophage phagocytosis (Fig. 1D).^11^ The density of CD47-self peptides directly influenced the duration of phagocytosis inhibition. Jalil et al. designed a soluble multivalent anti-CD47 peptide and observed that bivalent and tetravalent peptides enhanced macrophage phagocytosis of tumor cells compared to monovalent peptides.^135^ Importantly, systemic application of these peptides did not result in severe side effects, such as anemia.

### 5.2 Fcγ receptor

**Figure 5:**
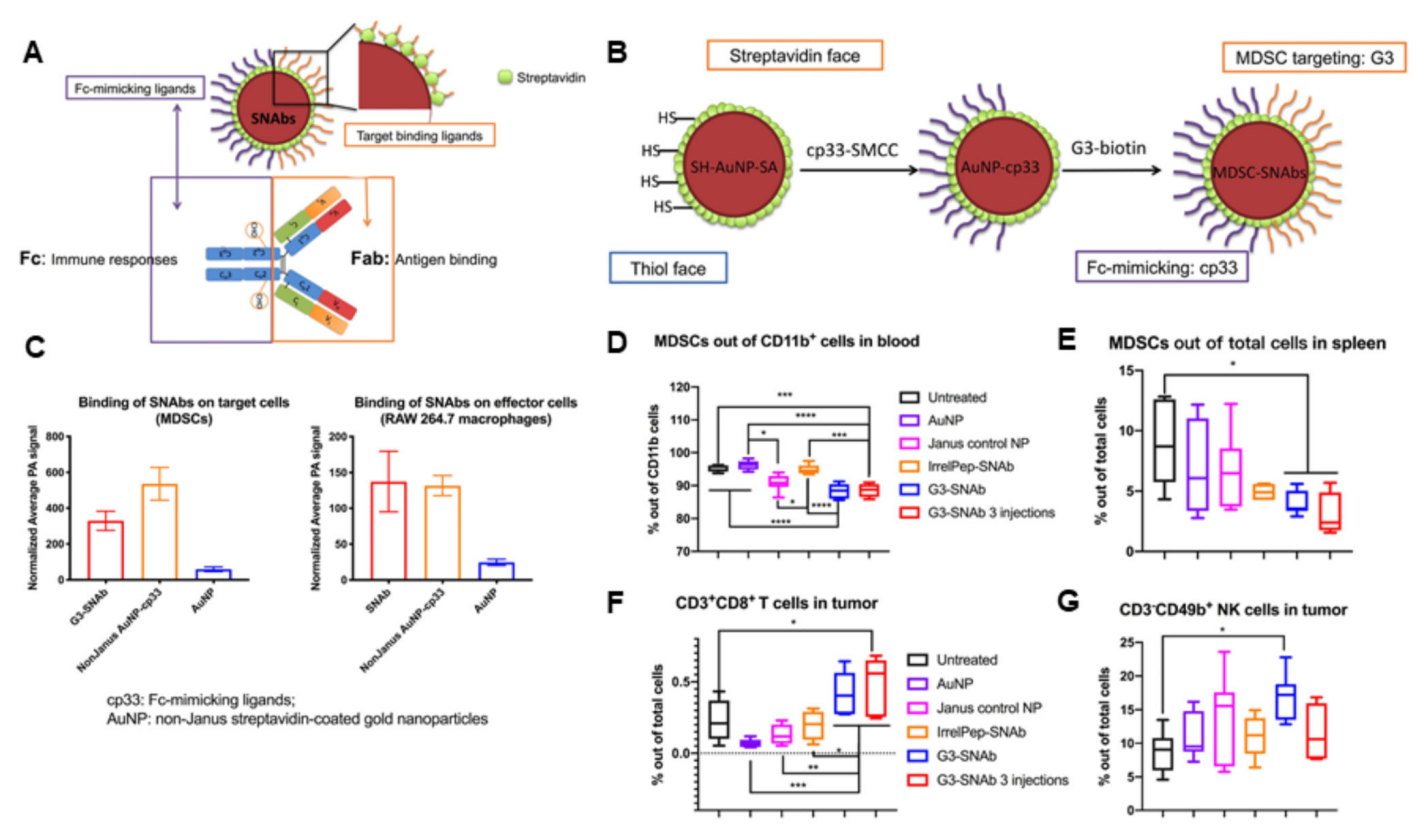
Bifunctional nanoparticles promoted phagocytosis of MDSCs by macrophages. (A) Schematic of synthetic nanoparticle-antibodies (SNAbs). (B) Schematic of MDSC-targeting SNAbs (G3-SNAbs). (C) Binding of SNAbs to effector cells and target cells. (D, E) G3-SNAbs cleared MDSCs within the blood (D) and spleen (E). (F, G) CD8^+^ T cells (F) and NK cells (G) were elevated by G3-SNAbs in tumors. A-G are adapted from Liu et al.^12^

While CD47-SIRPα signaling inhibit phagocytosis, FcγRs recognize the Fc segment of the antibody and cause macrophages to phagocytoze antibody-labeled cells.^136^ These two pathways work in tandem to regulate macrophage phagocytosis: the CD47-SIRPα pathway identifies normal cells, while the FcγRs pathway targets abnormal cells or pathogens marked by antibodies. According to Pacheco et al., the efficiency of macrophage phagocytosis of particles within the range of 0.5 µm to 2 µm was influenced by the density of the Fc ligands, whereas phagocytosis of particles larger than 2 µm primarily occured through non-specific mechanisms.^137^ Additionally, FcγR-mediated phagocytosis imposed specific requirements of the length of the ligand on the target particle.^138^ Kern et al. controlled ligand density and distribution on lipid-coated silica beads using DNA origami.^139^ Macrophages exhibited a preference for phagocytizing particles with ligands separated by less than 7 nm. High ligand density promoted FcγR clustering, phosphorylation of FcγRs, and activation of downstream Syk signaling. Considering the Fc fragment-mediated phagocytosis by macrophages, nanoparticles functionalized with F(ab')2 fragments of antibodies can effectively reduce particle clearance.^140^ Moreover, the Fc-mediated phagocytosis can be leveraged to design nanoparticles that enhance macrophage phagocytosis of target cells. Liu et al. developed Janus Au nanoparticles designed to direct macrophages to engulf antibody-labeled cells (Fig. 1D and Fig. 5A).^12^ One side of the nanoparticles was modified with the FcR ligand cp33, which facilitated macrophage phagocytosis by crosslinking FcγRs (Fig. 5B). On the other side, the nanoparticles were modified with the myeloid-derived suppressor cells (MDSC)-targeting peptide G3 (Fig. 5B). These bifunctional nanoparticles G3-SNAbs effectively bound to both effector cells (macrophages) and target cells (MDSCs) (Fig. 5C). Tumor-bearing mice treated with G3-SNAbs significantly reduced the amount of MDSC in the blood and spleen, (Fig. 5D, 5E) and increased the proportion of intratumoral CD8^+^ T cells and NK cells (Fig. 5F, 5G).

## 6 Natural killer cell surface receptors

**Figure 6:**
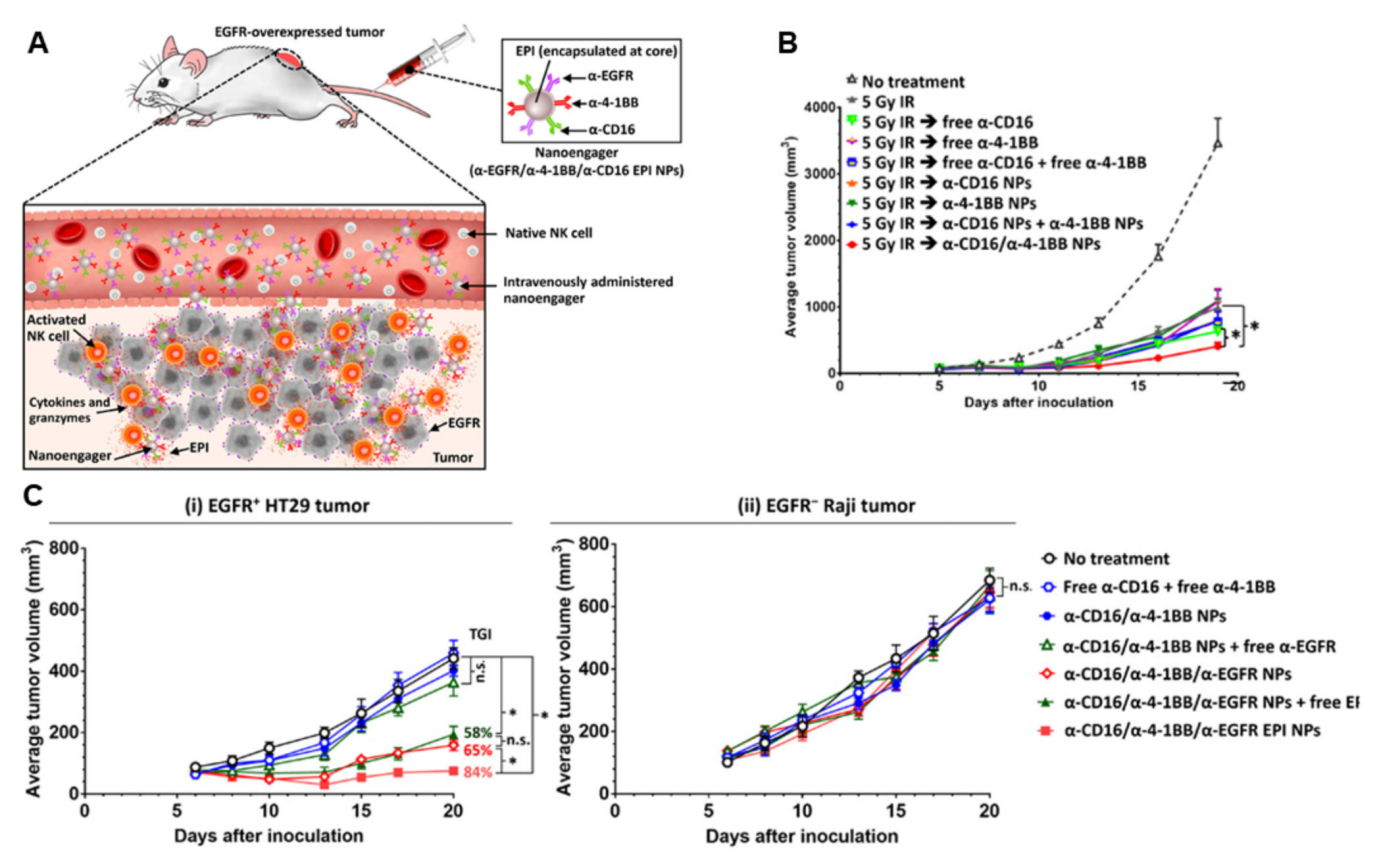
Trispecific nanoparticles promoted NK cell-mediated inhibition of EGFR-positive tumors. (A) Schematic of trispecific nanoparticle-mediated tumor suppression. (B) Trispecific nanoparticles synergistically inhibited B16F10 tumors with radiotherapy. (C) Trispecific nanoparticles specifically inhibited EGFR-positive HT29 tumors. A-C are adapted from Au et al.^146^. © The Authors, some rights reserved; exclusive licensee AAAS. Distributed under a CC BY-NC 4.0 license http://creativecommons.org/licenses/by-nc/4.0/. Reprinted with permission from AAAS.

Natural killer (NK) cells distinguish healthy cells by recognizing MHC I molecules on the cell surface. Typically, all nucleated healthy cells express MHC I, which triggers inhibitory receptors on NK cells.^141^ Cancer cells reduce their MHC I expression to evade T cell attacks. In such scenario, NK cells recognize cancer cells and eliminate them. Achieving full activation of NK cells relies on the assistance of activating receptors, such as CD16. CD16 is also known as FcγRIII that activates natural killer (NK) cells and mediates antibody-dependent cellular cytotoxicity (ADCC).^142^ CD16-Fc ligation enables NK cells to rivet target cells, thus secretiing granzyme and perforin to lyse target cells (Fig. 1E). The extensive receptor-ligand engagement between cells mediates the crosslinking of cell surface receptors and exerts activation or inhibition effects.^134,143^ CD16 crosslinking enhances NK cell degranulation and target cell lysis compared to NK cells without CD16 crosslinking.^144^ Bispecific and trispecific antibodies immobilized tumor cells induced CD16 crosslinking on NK cells upon contact with tumor cells and exhibited specific lytic activity.^142,145–147^ CD16 crosslinking was affected by ligand spacing, where increased ligand spacing hindered CD16 crosslinking and inhibited NK cell activation.^32^ Au et al. developed trispecific nanoparticles by incorporating anti-CD16 antibodies, anti-4-1BB antibodies and anti-EGFR antibodies onto polyethylene glycol-block polylactide-co-glycolide (PEG-PLGA) nanoparticles (Fig. 6A).^146^ Nanoparticles coated with anti-CD16/anti-4-1BB antibodies inhibited the growth of B16F10 tumors when combined with radiotherapy (Fig. 6B). Trispecific nanoparticles effectively suppressed EGFR-positive HT29 tumors (Fig. 6C). Furthermore, pH-dependent controllable release of encapsulated epirubicin (EPI) promoted the anti-tumor effect of the trispecific nanoparticles (Fig. 6C).

## 7 Conclusion

For in vivo applications of biomaterials designed to interact with immune cell surface receptors, the delivery of these materials is critical. In this review, we provide an overview of biomaterials spanning a wide size range, from the nano- to macro-scale, emphasizing that the size of the biomaterial is the primary determinant of the delivery route. For nanoparticles below 200 nm, delivery can be achieved through intravenous administration or local injections (e.g., subcutaneous, intramuscular or intro-lesion) depending on the target tissue and the specific properties of the biomaterials, including their stability in the bloodstream.^148^ Additionally, alternative routes such as inhalation may be employed to target the lung. Notably, oral delivery is a feasible approach, with potential applications in stimulating gut immunity or enabling absorption through the gut mucosa for systemic drug distribution. Conversely, for biomaterials of larger scales, ranging from micro- to macro-scales, the available delivery options are more limited. These biomaterials are predominantly administered through local injections or implantation, often in intra/peri-tumoral contexts. Notably, oral administration remains a possibility for larger biomaterials.

The territory of cancer immunotherapy has continuously expanded. As discussed in the current review, the combination of biomaterials and immunotherapy offers promising possibilites for the treatment of tumors. Biomaterials-based combination cancer immunotherapy has also been tested in clinical trials.^149^ Multiscale biomaterials are used to activate anti-tumor immunity, including but not limited to polymers, nanoparticles, and hydrogels. The materiels have been used to crosslink immune cell surface receptors for immune activation. In contrast to immunotherapeutic delivery strategies, the covalent linkage of biomolecules to biomaterials enables the interaction between materials and immune cell surface receptors, mimicking the multivalent cell-to-cell or cell-to-pathogen interactions. These approaches are highly tunable. For example, increasing the aspect ratio of nanoparticles increased the contact area between materials and cells.^68^ Semi-flexible rather than rigid biomaterials enabled efficient clustering of immune cell surface receptors.^30^ In addition, biomaterials can couple multiple biomolecules for the crosslinking of multiple surface receptors. It is crucial to deepen our understanding on cell-cell interations to develop more effective immunoengineering strategies. This understanding should consider the influence of mechanical factors, such as force and deformation, on these interactions and their impact on immune responses. Biomaterial-based cell surface receptor crosslinking strategies have the potential to significantly contribute to cancer immunotherapy.
